# Physical Stability Studies of Semi-Solid Formulations from Natural Compounds Loaded with Chitosan Microspheres

**DOI:** 10.3390/md13095901

**Published:** 2015-09-16

**Authors:** Niuris Acosta, Elisa Sánchez, Laura Calderón, Manuel Cordoba-Diaz, Damián Cordoba-Diaz, Senne Dom, Ángeles Heras

**Affiliations:** 1 Department of Physical Chemistry II, Faculty of Pharmacy, Institute of Biofunctional Studies, Complutense University, Paseo Juan XXIII nº 1, Madrid 28040, Spain; E-Mails: elisas01@ucm.es (E.S.); lcaldero@ucm.es (L.C.); 2 Department of Pharmacy and Pharmaceutical Technology, Faculty of Pharmacy, Institute of Industrial Pharmacy, Complutense University, Plaza Severo Ochoa s/n., Madrid 28040, Spain; E-Mails: mcordoba@ucm.es (M.C.-D.); damianco@ucm.es (D.C.-D.); sene_dom@hotmail.com (S.D.)

**Keywords:** chitosan, spray-drying, semi-solid formulation, antioxidant encapsulation

## Abstract

A chitosan-based hydrophilic system containing an olive leaf extract was designed and its antioxidant capacity was evaluated. Encapsulation of olive leaf extract in chitosan microspheres was carried out by a spray-drying process. The particles obtained with this technique were found to be spherical and had a positive surface charge, which is an indicator of mucoadhesiveness. FTIR and X-ray diffraction results showed that there are not specific interactions of polyphenolic compounds in olive leaf extract with the chitosan matrix. Stability and release studies of chitosan microspheres loaded with olive leaf extract before and after the incorporation into a moisturizer base were performed. The resulting data showed that the developed formulations were stable up to three months. The encapsulation efficiency was around 44% and the release properties of polyphenols from the microspheres were found to be pH dependent. At pH 7.4, polyphenols release was complete after 6 h; whereas the amount of polyphenols released was 40% after the same time at pH 5.5.

## 1. Introduction

Chitosan is a linear biopolyaminosaccharide obtained by alkaline deacetylation of chitin, which is the second most abundant polysaccharide, surpassed only by cellulose [1]. Chitosan possesses free amino and hydroxyl groups, which facilitate its cross-linking reaction to form chitosan cross-linked microspheres [2]. The use of chitosan for the encapsulation of active components has attracted interest in recent years due to its mucoadhesiveness, non-toxicity, biocompatibility, and biodegradability. Antibacterial and moisturizing properties and other beneficial features of chitosan, especially in emulsions and topical gels for application in biomedicine and cosmetics, have been extensively described [3,4]. The cationic character of chitosan, along with the presence of reactive functional groups, provides particular possibilities for utilization in controlled-release technologies [5]. Numerous varieties of chitosan can be obtained due to the different chitin sources and to differences in deacetylation conditions. The presence of free amino groups is responsible for the interaction of chitosan with biological systems, and the distribution of deacetylated groups along the chitosan molecule may regulate these interactions. The wide variety of products that can be obtained as a result of the chemical modification of chitosan can enhance its already valuable properties. A wise selection of the suitable type of chitosan can lead to the development of customized delivery systems. Chitosan-based modified release systems may prolong the duration of active agent activity, improving its efficiency and reducing side effects under desired conditions, through the modification of its pharmacokinetic characteristics. It has been found that chitosan has a slight positive charge, which makes it soluble in acid or neutral solutions depending on the pH and degree of deacetylation. This feature also provides bioadhesive properties because chitosan can ionically link to mucosal surfaces. Due to this physical property, chitosan facilitates the transport of polar active ingredients through the epithelial surfaces. The benefits of encapsulating active agents in a polymer matrix include their protection from the surrounding medium or processing conditions and the possibility of controlling release [6]. The combination of chitosan, a natural polymer, with other compounds such as antioxidants generates a new system joining the properties of both components, which improves the stability of the antioxidants and controls their pharmacokinetic properties [7].

The olive leaf extract (*Olea europea* L.) is rich in phenolic compounds, with strong antioxidant activity. The most abundant phenolic compounds available in the olive leaf are tyrosol and hidroxytyrosol. Hydroxytyrosol is known for its capacity to stop oxidative stress and neutralize free radicals [8]. Due to its beneficial properties and abundance, olive leaf extract has been chosen as a source of antioxidant compounds for our study.

The popularity of natural ingredients in cosmetics is increasing in the market to satisfy the needs of consumers. The possibility of allergies and skin irritations due to synthetic preservatives (such as parabens and stabilizers) has not yet been fully tested for long-term consequences on the health of consumers [9]. Some vegetable oils containing essential fatty acids (EFAs) have proven to be of great use in the formulation of cosmetics, either as a component of high potential activity or as precursors for the synthesis of novel compounds. It is well described that EFAs are easily integrated into the hydro-lipid layer structures of the skin and they provide nourishing, moisturizing, and protective properties. Apart from their moisturizing, smoothing, and anti-inflammatory effects, these products reduce skin aging with their antioxidant and stabilizing action on the cellular membranes [10].

The human skin is an important target for drug delivery due to the inherent advantages of the transdermal route in the treatment of some pathologies. Many formulations have been designed for transdermal delivery of different substances using different strategies, in spite of the fact that one of the most important functions of the skin is as a barrier against xenobiotic agents [11].

In this study, different topical formulations including essential oils were designed in order to examine its stability over time under different conditions and to study their beneficial effects. The work described here was complemented with the use of chitosan microparticles loaded with olive leaf extracts to examine its influence on the organoleptic characteristics of the emulsions and to enhance their antioxidant effects over the skin in terms of the release of antioxidant compounds.

The main aim of this study is focused in the incorporation of chitosan microparticles loaded with olive leaf extracts in different topical formulations for cosmetic use. The obtained microspheres were previously characterized in terms of morphology, zeta potential, particle size, drug-polymer interactions, encapsulation efficiency, and *in vitro* release profile. Physicochemical and stability studies of the different formulations obtained and the release profiles of the active ingredient were also carried out over time.

## 2. Results and Discussion

### 2.1. Physico-Chemical Characterization of Chitosan Batches

The average molecular weight and deacetylation degree of chitosan are two of the main characteristics that affect the physico-chemical and biological properties of chitosan and its derivatives [12]; a structural analysis and determination of this parameter became necessary in order to establish a relationship to the functional properties. The molecular weight average was determined by gel permeation chromatography, obtaining a value of 84,500 Da; this value indicates the presence of relatively long polymer chains that generate potential crossovers that can provoke a decrease in water solubility [13]. The degree of deacetylation was determined by nuclear magnetic resonance (^1^H-NMR), obtaining an average value of 84%, which denotes the presence of a high percentage of NH_2_ protons with respect to the acetamides groups. It is well known that the molecular weight is related to the acetylation degree. In this way, an increase of the molecular weight is associated to an increase of the acetylation degree. Chitosan has a deacetylation degree with high value, which indicates a high solubility of the chitosan in an acidic medium due to the protonation of its amino groups.

### 2.2. Antioxidant Activity and Total Amount of Polyphenols in the Olive Leaf Extract

The values of the antioxidant activity of the olive leaf extract were obtained by comparing the absorbance change at 595 nm in test reaction mixtures with those containing ferrous ions at a certain known concentration using a calibration curve. The antioxidant activity of the olive leaf extract selected for our study was 73.34 ± 0.7 mM (mean ± SD of six determinations).

The Folin-Ciocalteu method [14] was used to measure the total amount of polyphenols presents in the olive leaf extract. Absorbance of the developed coloration was measured at 765 nm against a blank sample. Gallic acid was used as the standard and the results were expressed as g/L of gallic acid equivalents. The value of the total amount of polyphenols present in our olive leaf extract was 9.28 ± 0.03 g/L.

These results correlate quite well to those obtained by other authors [15] in terms of total phenol values that were congruent with FRAP and Folin assays: antioxidant values with *R*^2^ values of 0.932 and 0.736, respectively. The results suggested that the olive leaf extract selected for our study constitutes a natural alternative to synthetic antioxidants due to the high amount of antioxidants present within its composition, being a promising natural alternative for the prevention of oxidative damage.

### 2.3. Physico-Chemical Characterization of Chitosan/OLE Microspheres

**Figure 1:**
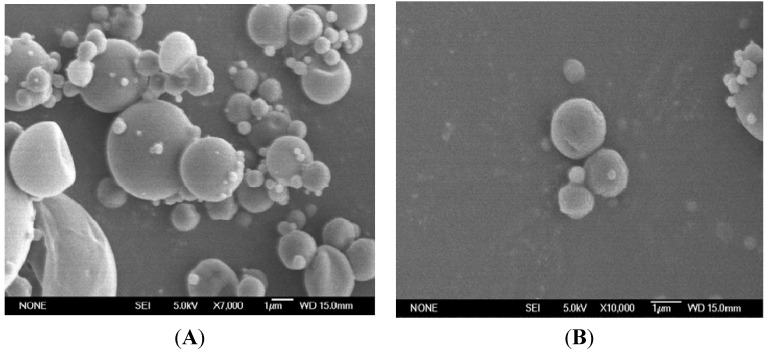
SEM micrographs of chitosan microspheres loaded with olive leaf extract. (**A**) Microspheres at 7000 magnification and (**B**) microspheres at 10000 magnification.

Chitosan microparticles were prepared by spray-drying, a technique commonly used in pharmacy to produce a dry powder from a liquid phase [16,17]. The yield of the spray-drying process was around 70%. The morphology of the resulting chitosan microspheres loaded with olive leaf extract is shown in Figure 1A,B. All prepared microparticles were spherical and showed a smooth surface without any indentations or irregularities. Particle size analysis revealed a highly dispersed distribution, although it could be observed that the vast majority of the studied particles showed a diameter below 5 μm.

The total polyphenol content in the obtained chitosan microspheres was measured and the encapsulation efficiency was near 44%. Therefore, it was proven that our chitosan microspheres maintained the stability of the polyphenols over time and constitute a good vehicle for the encapsulation of these compounds, which corroborates the findings of other authors for this kind of system e.g., [5], who also observed that the encapsulation of olive leaf extract by spray-drying did not lead to the inactivation of the polyphenolic compounds.

**Figure 2:**
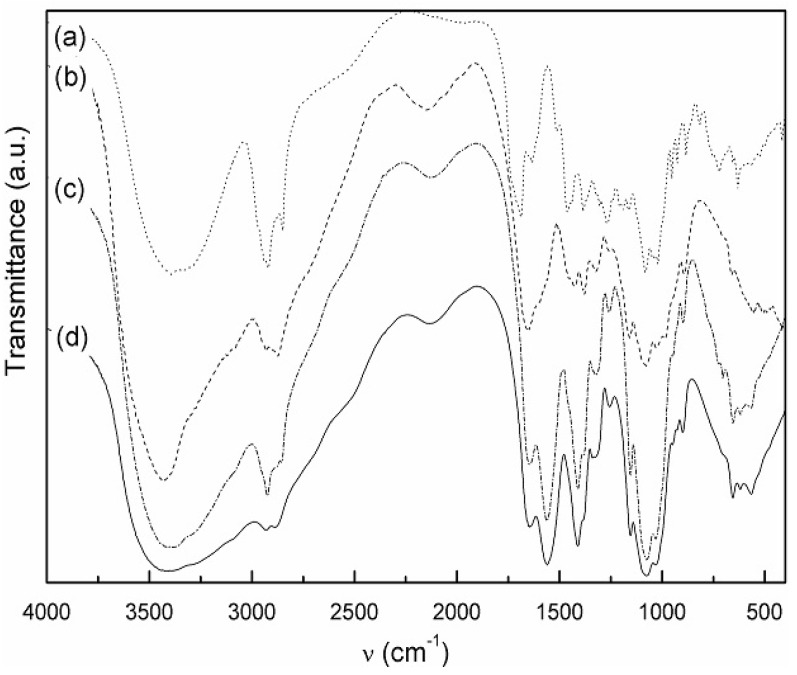
FTIR of olive leaf extract (**a**); chitosan (**b**); olive leaf extract loaded microspheres (**c**); chitosan microspheres (**d**).

The chitosan FTIR spectra shown in Figure 2 exhibited distinctive absorption bands at 1658 cm^−1^ (Amide I), 1595 cm^−1^ (-NH_2_ bending) and 1320 cm^−1^ (Amide III). The absorption bands at 1154 cm^−1^ (anti-symmetric stretching of the C-O-C bridge), 1080, and 1030 cm^−1^ (skeletal vibrations involving the C-O stretching) are characteristic of its saccharide structure [18,19]. It can be also observed that in the spectrum of chitosan microspheres the amine band shifted to 1560 cm^−1^. This shift has been previously reported for chitosan acetate films [20]. The intensity of the band at 1560 cm^−1^ was increased by the contribution of the asymmetric COO^−^ stretching vibration, resulting from the carboxylate groups of the acetate ion. The symmetric COO^−^ stretching band can be observed at 1406 cm^−1^.

The IR spectrum of the chitosan microspheres loaded with olive leaf extract was found to be very similar to the spectrum obtained from the unloaded microspheres. The main difference between them lies in the presence of a stronger C-H stretching absorption band at 2919 cm^−1^ in the loaded microspheres. This band was also observed in the spectrum of the olive leaf extract shown in Figure 2.

The above results corroborate the presence of olive leaf extract in the microspheres. However, from the analysis of IR spectra no specific interactions between the extract and chitosan were evident.

**Figure 3:**
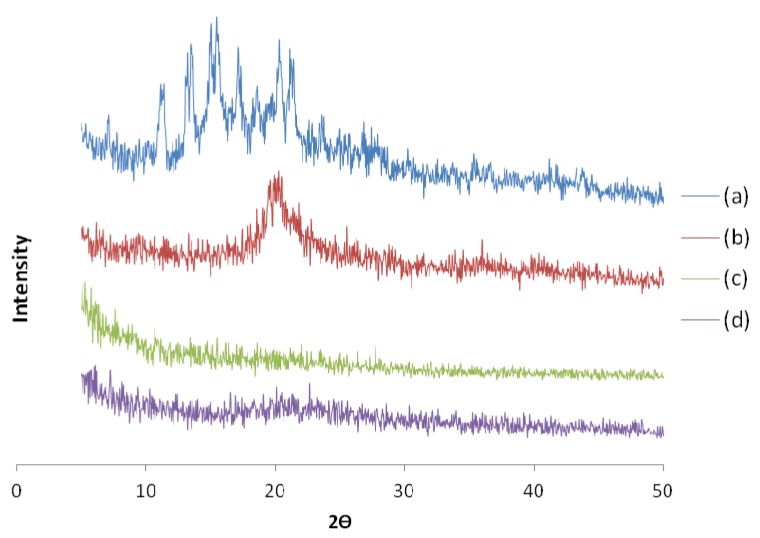
XRD of olive leaf extract (**a**); chitosan (**b**); olive leaf extract loaded microspheres (**c**); and chitosan microspheres (**d**).

In order to ascertain the possible interaction of polyphenols and the polymer, some experiments were performed. XRD plays a prominent role in the characterization of polymeric particles because it is able to provide structural information on the dispersed particles [21]. The X-ray diffractions of chitosan, empty microspheres, olive leaf extract, and OLE/chitosan microspheres are shown in Figure 3. The diffractogram from the chitosan sample showed a weak peak at 2θ of 10° and a more intense peak at 2θ of 20°, caused by diffraction from (020) and (110) planes of the crystalline lattice with inter-planar distances of 0.88 nm and 0.45 nm [22]. The diffractogram of the chitosan sample indicated that the crystalline phase coexists with a considerable amount of amorphous phase [23]. Comparing the diffractograms of chitosan and chitosan microspheres, it can be seen that the crystallinity decreases with the formation of chitosan microspheres. On the other hand, olive leaf diffractograms showed various peaks not present in the diffractogram of OLE/chitosan microspheres, which indicated that the olive leaf extract is molecularly dispersed within the polymer matrix. It was proven that the incorporation of the olive leaf extract into the chitosan microspheres did not affect the amorphous structure of the particles, which agrees with findings reported by Yenilmez *et al.* [24].

The surface charge of the microspheres was measured to evaluate the stability of the suspended particles. The positive surface charge of the chitosan microspheres loaded with olive leaf extract promotes mucoadhesion and hinders aggregation, which results in particles with a good stability. The zeta potential of microspheres was +47.58 ± 1.48 mV (mean ± SD of three determinations). These delivery systems have mucoadhesive potential and absorption enhancement properties [25].

### 2.4. In Vitro Release Profile of Olive Leaf Extract Loaded Microspheres

The objective of the encapsulation of antioxidants is their release into the skin to prevent damage caused by free radicals and to help reversing the signs of aging. The extract of olive leaves is rich in polyphenols and these have a high antioxidant power.

**Figure 4:**
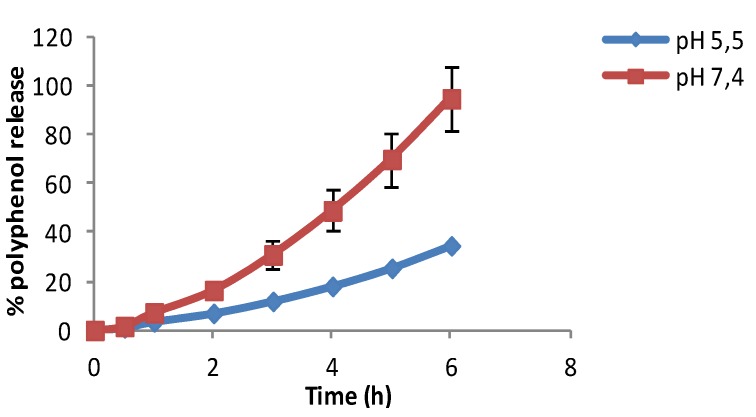
*In vitro* release profile of polyphenols of chitosan microspheres loaded with olive leaf extract in buffers pH 5.5 and pH 7.4.

Although the pH of the skin is 5.5, the pH of cosmetic formulations can range between 5.5 and 7 [26]. The release studies of chitosan microspheres loaded with olive leaf extract were performed in two buffers with different pH values (pH 5.5 and 7.4). Release profiles at pH 5.5 and 7.4 are shown in Figure 4.

The release profiles of chitosan microspheres loaded with olive leaf extract were investigated *in vitro* at 37 °C. As can be seen in Figure 4, a complete release of polyphenols was achieved after 6 h at pH 7.4, whereas at pH 5.5 the maximum release was near 35% of the initial amount of polyphenols. It can be seen that the differences of the release rate at different pH values were clear after the first 2 h. It was found that the release rate at a pH of 7.4 doubled the one obtained at a pH value of 5.5. According to the figures, it can be concluded that the polyphenols’ release was pH dependent [27]. The low solubility of chitosan with a smaller number of acetyl groups is caused by difficulties in attaching protons to amino groups that are close to protonated amino groups, due to electrostatic repulsive forces. The diffusion of the polyphenols was enhanced at high pH due to the deprotonation of chitosan, resulting in an improved release. So we can conclude with these results that an effective release was obtained at a pH of 7.4. These results also confirm the antioxidant profile release, corroborating the previous findings of other authors [18] who observed that the encapsulation of OLE by spray-drying did not lead to an inactivation of the polyphenolic compounds.

### 2.5. Physico-Chemical Characterization of Semi-Solid Formulations

A physicochemical characterization of the three semi-solid moisturizing formulations was performed before carrying out the *in vitro* release assays, in order to measure their extensibility, penetrometry, and pH. The pH was around 5 in formulation B and C. This pH value was conditioned mainly by the essential oils present in their composition. Emulsion A had a higher pH value, caused by a greater amount of water in its composition. The extensibility coefficient turned out to be 2.000 mm^2^ for formulations B and C. In formulation A, the extensibility was 2.376 mm^2^ due to the higher amount of water, resulting in a reduction of the consistency. Formulations B and C showed penetrometry values of 3.300 g·cm/s². This parameter resulted to be only 2.600 g·cm/s² for formulation A. No significant data differences among the different formulations were observed in extensibility and penetrometry tests.

Under normal conditions, it is accepted that skin pH ranges between 5 and 6. Hence, a topical formulation should have a pH near that range to avoid irritability and problems of tolerance. For this reason, pH was one of the parameters studied to evaluate the physical stability of our emulsions. It was found that the pH values of all formulations ranged between 5 and 6 depending on storage time. The stability studies were performed comparing, among other parameters, the controlled pH variations along different times. The results showed no significant differences between the studied formulations.

All the formulations were also tested from an organoleptic point of view due to the big importance of these properties for consumers, especially in cosmetics. The physical appearance of the emulsion formulations kept at 25 °C was examined visually at different times. In this connection, the formulations prepared were found to be stable in structure, without any change in physical appearance, and were found to keep their clear, transparent, and uniform appearance. No phase separation was visually observed in formulations after stored for three months at the same conditions.

**Figure 5:**
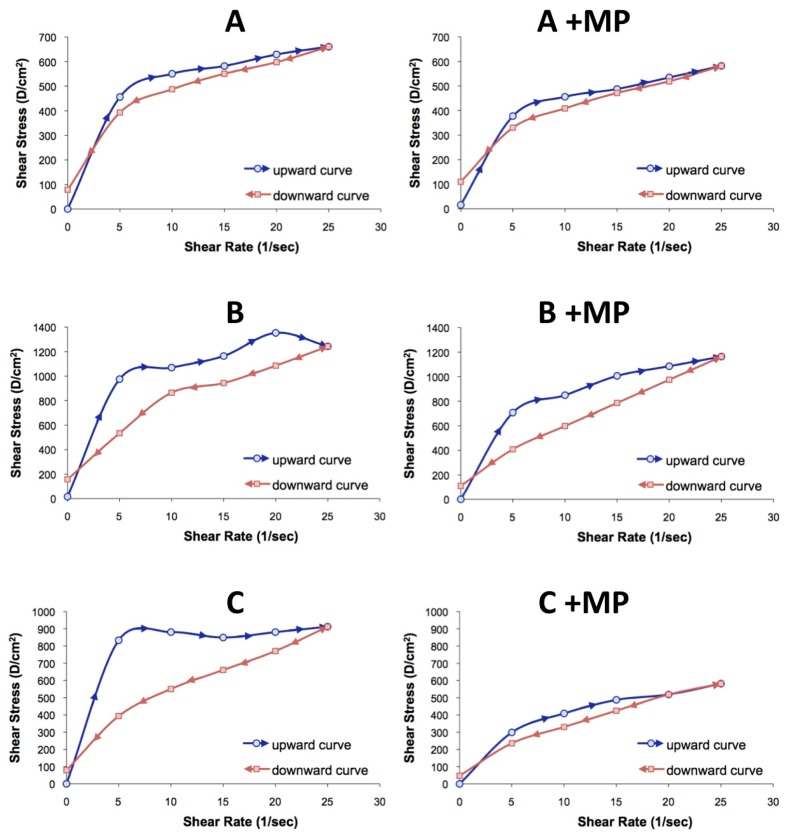
Rheological behavior of the semi-solid formulations with increasing shear rate. (**A**–**C**) Formulations without microspheres and A + MP, B + MP, and C + MP formulations with microspheres.

Viscosity data and rheograms are shown in Table 1 and Figure 5A–C. Typical flow curves for semisolid formulations with or without microspheres as a function of ascending-descending shear rate sweep are shown in Figure 5. In all experiments, shear stress increased to a maximum value at shear rate values near 25 s^−1^, then dropped off dramatically. These results suggest that structural disorganization occurs within the moisturizer base [28]. Viscosity values decreased in all experiments when the shear rate increased; this behavior is defined as pseudoplasticity or shears thinning, and it is indicative of structural breakdown. As can be seen in Figure 5, different behavior can be observed comparing the upward and downward curves of the rheograms, due to a delay in the reorganization of the internal structure of the semi-solid formulation. This phenomenon is known as thixotropy and can be associated with a decrease in viscosity due to a structural disorder after a continuous increase of the shear rate for a certain period of time [29].

**Table 1:** Viscosity data of the different formulations with and without microspheres.

	Viscosity V (Pa·s)	Thixotropy T (Pa)
Formulation		
A	3.146	8.65
A + MP	2.674	5.64
B	6.763	46.27
B + MP	5.426	37.62
C	4.404	47.58
C + MP	2.595	9.17

It was observed that the incorporation of chitosan microspheres into the semisolid formulations provoked significant changes over the rheological behavior. Formulations with a pseudoplastic flow produce a coherent film covering the skin surface, and this is important for a better antioxidant protection of the skin surface [30]. The results suggest that the incorporation of the microspheres into the semi-solid formulation produces changes in the internal structure of the formulation, modifying the apparent viscosity and reducing the thixotropy. These findings suggest that the inclusion of the microspheres loaded with olive leaf extract within the oil phase can modify the semi-solid structure.

Thixotropy is desirable in topical formulations because it helps to maintain the suspending components’ stability; moreover it can influence the active substances’ release to the skin due to the structural disarrangement of the system, where the active substances’ diffusion is facilitated. Although the thixotropy and viscosity values decreased for formulations with added microspheres, the rheograms obtained showed no instability signals. These results showed that these formulations could be considered stable comparing the rheological properties.

Likewise, a total bacteria recount was performed just after the elaboration of the formulations to test the possible errors in the manipulation, obtaining a negative result.

### 2.6. In Vitro Release Profile of Semi-Solid Formulations

**Figure 6:**
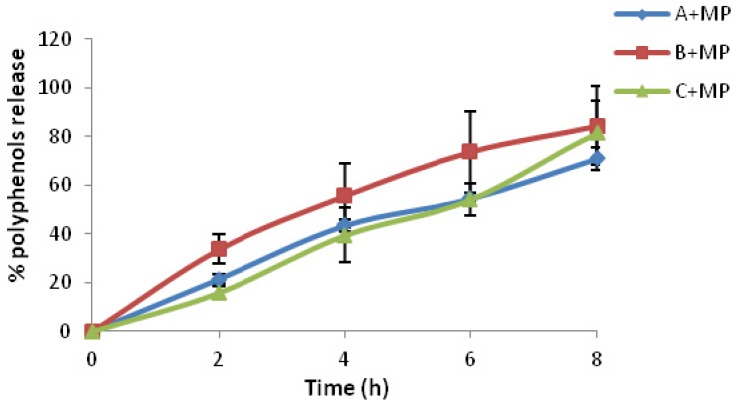
*In vitro* release profile of three different moisturizer bases with chitosan microspheres loaded with olive leaf extract at time 0.

The results in Figure 6 showed that the release of antioxidants from chitosan microspheres loaded with olive leaf extract after 8 h ranged from 60% to 80% at time 0 (just after elaboration of formulations) depending on the formulation. The results indicated that the release of the polyphenols of the olive leaf from chitosan microspheres varied with the time. Moisturizer bases A + MP released 60% of the total polyphenols after 8 h, whereas formulations B + MP and C + MP released around 80% of polyphenols at 8 h; these two moisturizers are rich in vegetable oils. Moisturizers with more vegetable oils in their composition had a higher release just after elaboration.

**Figure 7:**
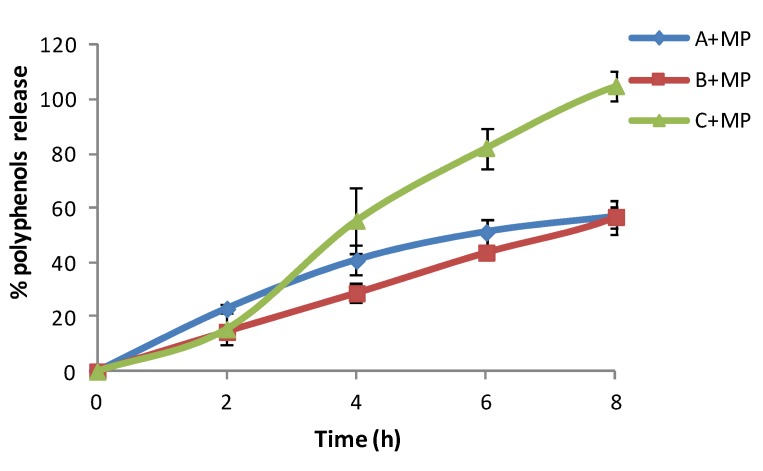
*In vitro* release profile of three different moisturizer bases with chitosan microspheres loaded with olive leaf extract stored for three months at 25 °C.

After 3 months of storage at 25 °C, formulation C + MP showed the highest percentage of polyphenols release (Figure 7). This might be due to the fact that the composition of this cream is rich in fatty oils and the emulsion is less stable, which can probably lead to a slight breakdown of the emulsion, or to the fact that fatty oils interfered with the measurements. The olive leaf extract release increases proportionally over time.

**Figure 8:**
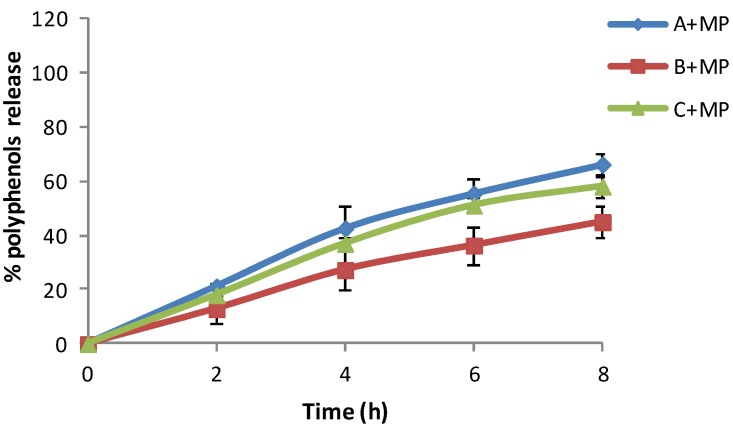
*In vitro* release profile of three different moisturizer bases with chitosan microspheres loaded with olive leaf extract stored for three months at 4 °C.

After 3 months of storage at 4 °C, formulation A + MP showed the higher percentage of polyphenol release (Figure 8). This might be due to the preservation of the formulation in low temperatures. The olive leaf extract release increases proportionally over time.

An individual analysis of each formulation at different storage temperatures evidenced some particularities.

**Figure 9:**
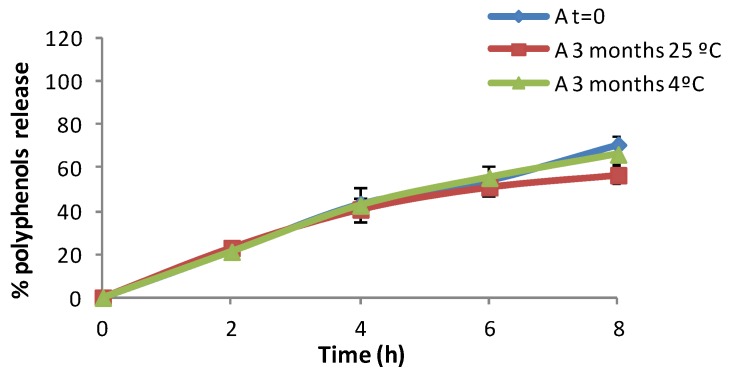
*In vitro* release profile of Formulation A + MP.

Figure 9 shows the *in vitro* release profile of Formulation A + MP stored at three temperatures for different lengths of time. In this formulation the percentage of *in vitro* release is maintained for long periods of time. The microspheres and the composition of the formulation are stable after three months at different storage temperatures.

**Figure 10:**
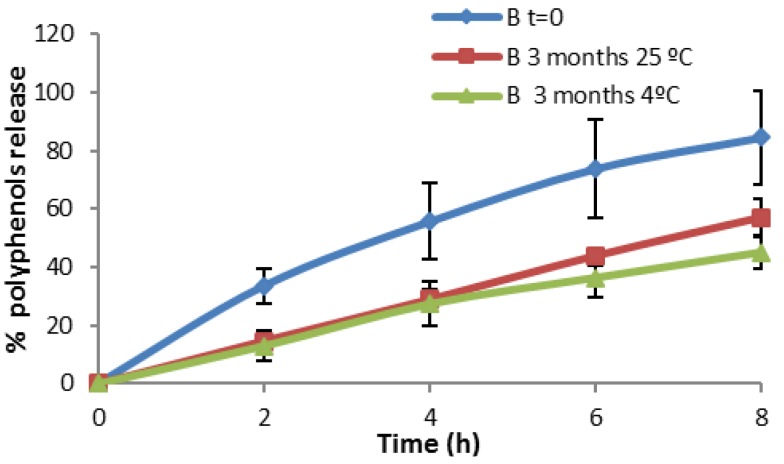
*In vitro* release profile of Formulation B + MP.

**Figure 11:**
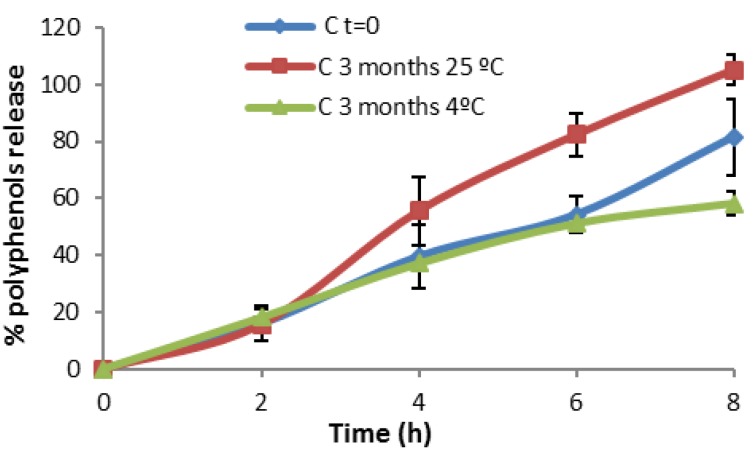
*In vitro* release profile of Formulation C + MP.

Figure 10 shows that at the initial time of the elaboration, the *in vitro* release of microspheres in formulation B + MP is more effective than that obtained after three months. Here, the temperature affects the physical stability of the formulation. It was found that the physical stability was improved by refrigerating the formulation (storage at 4 °C). Formulation C + MP in Figure 11 showed a higher release at high temperatures. This could be due to the breaking of the phases of the emulsion, resulting in the detection of polyphenols in the spectrophotometer. At time 0 (just after elaboration) the percentage of *in vitro* release is higher than after three months stored at 4 °C. This result is remarkable as the release was expected to remain more stable after storage in the fridge than after storage at room temperature. After comparing the *in vitro* release at different temperatures, we concluded that storage at 4 °C is the best method for maintaining the stability of the present formulations.

The encapsulation of olive leaf extract into chitosan microspheres aimed to protect the polyphenols from degradation and to control their release to obtain a longer lasting effect. It is clear that formulations A + MP and B + MP appeared to have the most promising results if we compare the release profiles of the different formulations.

A comparison of the different permeability values of the formulations is represented. The permeability coefficients for formulation A + MP and B + MP follow the same pattern: at time 0 the permeability coefficients are the highest (0.0073 cm/h and 0.0044 cm/h, respectively); these values decreased after 3 months of storage at 25 °C (0.0034 cm/h and 0.0022 cm/h, respectively). This decrease was also found under storage conditions of 4 °C (0.0043 cm/h and 0.0038 cm/h, respectively). This decrease is bigger for formulation A. After 3 months at 4 °C permeability, constants are higher than after 3 months at 25 °C, which is normal as storage in the fridge is considered to be better for the stability of the formulation. In contrast, Formulation C + MP showed different results: compared to time 0 (0.0065 cm/h), the permeability coefficient increased slightly after 3 months at 25 °C (0.0067 cm/h) and decreased after 3 months at 4 °C (0.0055 cm/h), which can be associated with a degradation process. The permeability coefficients obtained in these experiments gave an estimation of the release of polyphenols from the vehicle into the surface structures of the skin, providing an idea of the permeability of polyphenols through the skin.

Chitosan microspheres loaded with olive leaf extracts enhance the antioxidant effect of the polyphenols present in the moisturizer base. The chitosan microspheres have greater facility to diffuse into the medium and the release of the polyphenols was improved, enhancing the desired effect. Both the polyphenols present in vegetable oils and the polyphenols from the microspheres loaded with olive leaf extracts add value to the moisturizer base by providing antioxidant activity; this plays an important role in skin damage caused by oxidizing agents that are good regenerators and effective for reconstructing skin.

## 3. Experimental Section

Chitosan was obtained from InFiQuS S.L. The degree of deacetylation was 84% and the molecular weight (Mw) was 84.5 KDa. Olive leaf extract was provided by the University of Cordoba (Research group FQM-227, Spain). Formulation ingredients (calendula oil, jojoba oil, olivem^®^ 1000, glycerol stearate, xanthan gum, candy dye, eucalyptus water, sodium stearoyl lactylate, beeswax, shea butter, roses water, avocado oil, and chlorophyll dye) were purchased from Aromazone and were of pharmaceutical quality. All other reagents were commercially available and used as received.

### 3.1. Physico-Chemical Characterization of Chitosan

#### 3.1.1. Determination of Molecular Weight by Size Exclusion Chromatography

SEC-HPLC was performed in a Waters 625 LC System pump equipped with an Ultrahydrogel (Waters, i.d = 7.8 mm l = 300 mm) column thermostated at 35 °C. Waters 2414 differential refractometer and evaporative light scattering (ELS Waters 2424, Milford, MA, USA) were connected online. A 0.15 M ammonium acetate/0.2 M acetic acid buffer (pH 4.5) was used as eluent. Analytical samples were dissolved in the buffer solution (0.05%, w/v) and filtered through a 0.45 μm pore size membrane (Millipore Corporation, Beverly, MA, USA) before injection of aliquots of 20 μL. The flow rate was 0.6 mL/min. The Mw of the different fractions were obtained from the SEC profiles by extrapolation in a calibration curve using different known Mw chitosans as standards.

#### 3.1.2. Determination of Acetylation Degree by Nuclear Magnetic Resonance (^1^H-NMR)

Structural characterization of chitosan samples was carried out using nuclear magnetic resonance spectroscopy (^1^H-NMR). Samples were dissolved in a DCl/D2O 1% (w/v) mixture and placed in 5 mm NMR tubes. TSP (sodium 3-(trimethylsilyl) propionate-d4) was used as an internal standard. The measurements in the experiments were performed on an AMX 500 spectrometer (Bruker Ettinglen, Germany). The acquisition conditions were: 2s number of scans 128 for ^1^H-NMR experiments and a frequency of 125.77 MHz.

### 3.2. Antioxidant Activity and Total Amount of Polyphenols in the Olive Leaf Extract

Antioxidant activity was determined by the ferric reducing/antioxidant power (FRAP) assay at 595 nm. The method is based on the reduction of a ferric 2, 4, 6-tripyridyl-s-triazine complex (Fe^3+^-TPTZ) to the ferrous form (Fe^2+^-TPTZ) [31,32] using trolox (a water-soluble vitamin E analogue) as the standard.

Total polyphenols content was spectrophotometrically quantified at 750 nm using the Folin–Ciocalteu reagent [14] and gallic acid as the standard. GBC UV/Vis 920 spectrophotometer (GBC Scientific Equipment PTY LTD, Melbourne, Victoria, Australia) was used in both methods.

### 3.3. Preparation of Chitosan Microparticles Loaded with Olive Leaf Extract

Microspheres were prepared by the spray-drying technique [33]. Briefly, an accurately weighed amount of chitosan was dissolved in 0.2% acetic acid aqueous solution to obtain a concentration of 0.83% w/v. Olive leavf extract (OLE) (10% w/v) mixed with ethanol (96%) was added dropwise to the acidic chitosan solution, under mild agitation. The resulting solution was spray-dried using a mini spray-dryer (Büchi Mini Spray-Dryer B-290, Switzerland) with an inlet temperature of 160 °C and an outlet temperature of 80 °C. The effect of the encapsulating system on the active compound stability and its release profiles was analyzed.

### 3.4. Physico-Chemical Characterization of the Chitosan Microparticles

#### 3.4.1. Estimation of the Encapsulation Efficiency of Polyphenolic Compounds

Five milligrams of chitosan microspheres loaded with olive leaf extracts were incubated with 1 N hydrochloric acid solution for 24 h. The amount of encapsulated olive leaf extract was estimated spectrophotometrically by the Folin-Ciocalteu method, expressed as a loading percent in terms of the amount of gallic acid in 100 g of microspheres.

#### 3.4.2. Evaluation of the Microparticles’ Morphology by Scanning Electron Microscopy

The shape and surface of the obtained microparticles were studied by scanning electron microscopy (SEM). The samples were examined using a scanning electron microscope JEOL JSM-6400 (JEOL, Tokyo, Japan). Microparticle surfaces were sputter-coated with a thin layer of gold for SEM visualization. The SEM images were taken by applying an electron-accelerating voltage of 15 kV.

#### 3.4.3. Drug-Polymer Interactions

##### Infrared Spectroscopy FT-IR

The FT-IR spectra were obtained using a Magna-IR 750 (Nicolet) spectrometer with a resolution of 4 cm^−1^ (50 scans, resolution 4.000, wavenumber range 4000–500 cm^−1^). X-ray diffraction patterns were obtained using an X-ray diffractometer (PHILIPS X0PERT SW) equipped with a copper anode. The samples were scanned continuously from 0° to 50° (2θ) at 45 kV and 40 mA.

#### 3.4.4. Zeta Potential Measurements

The Z potential value of the chitosan microspheres in aqueous solution was measured using a Nano-Zetasizer system (Malvern Instruments, Herrenberg, Germany). Every measurement was carried out in three serial measurements. Electrophoretic mobility, Z-potential, average size, and polydispersity index were obtained for each batch of microparticles.

#### 3.4.5. *In Vitro* Release Profile of Olive Leaf Extract Encapsulated in Microspheres

The release of olive leaf extracts expressed as the total amount of polyphenols under predetermined conditions was investigated. Microspheres (50 mg) (Mw cut off 12,000 Da, Sigma-Aldrich, Madrid, Spain) were suspended in 5 mL of PBS solutions (Sigma-Aldrich, Madrid, Spain) with different pH values (pH 5.5 and pH 7.4), inside a cellulose dialysis bag. The dialysis bag was immersed in 50 mL of phosphate buffered saline (PBS) release medium with different pH values (pH 5.5 and pH 7.4) at 37 °C and 100 rpm (Rotabit horizontal shaker, Selecta, Barcelona, Spain). One-milliliter samples were taken at specific time intervals and replaced with fresh medium. The release of the polyphenols was quantified using the Folin-Ciocalteu method. All the experiments were carried out in triplicate.

### 3.5. Design of Semi-Solid Formulations Containing Chitosan Microspheres

Microspheres were incorporated into three different base formulation prepared by o/w and w/o emulsions. The water phase and oil phase were heated separately up to 60 °C until homogenization of all the components. Microspheres were added into the oil phase at a concentration of 0.2% (w/w). Finally, the water phase was mixed with the oil phase and vigorously stirred together. For control purposes, a moisturizer sample without added microspheres was also prepared. Samples with and without microspheres were analyzed. Compositions of the semi-solid formulations are shown in Table 2.

**Table 2:** Compositions of the semi-solid formulations.

Formulation	Oil Phase Ingredients	Water Phase Ingredients
A(o/w emulsion)	0.8% (w/w) calendula oil 2.5% (w/w) jojoba oil 13% (w/w) olive oil 1.6% (w/w) olivem^®^ 1000 (emulsifier) 2.5% (w/w) glycerol stearate (emollient) 0.35% (w/w) xanthan gum (thickener) 0.25% (w/w) candy dye 0. 2% (w/w) OLE/chitosan microspheres	52.4% (w/w) purified water 26.4% (w/w) eucalyptus water
B (o/w emulsion)	6.5% (w/w) olive oil 3.7% (w/w) jojoba oil 5.5% (w/w) calendula oil 4.5% (w/w) sodium stearoyl lactylate 0.9% (w/w) beeswax 8% (w/w) glycerol stearate (emollient) 1.7% (w/w) shea butter 0. 2% (w/w) OLE/chitosan microspheres	46% (w/w) purified water 23% (w/w) roses water
C (w/o emulsion)	9.7% (w/w) jojoba oil 29% (w/w) avocado oil 5.5% (w/w) beeswax 3.2% (w/w) sodium stearoyl lactylate (emulsifier) 4.3% (w/w) shea butter 0.10% (w/w) chlorophyll dye 0. 2% (w/w) OLE/chitosan microspheres	32% (w/w) purified water 16% (w/w) eucalyptus water

#### Physico-Chemical Characterization of Semi-Solid Formulations

The organoleptic properties including general appearance, consistence, suffusing onto the skin, absorption, homogeneity of the formulation, absence of phase separation, and creaming and oily sensation on the skin were evaluated. Furthermore, certain changes in the product emerging in short-term storage such as color and odor were observed.

Rheological characterization of all the developed semi-solid formulation was carried out. Flow properties were studied using a previously calibrated Brookfield rotational viscosimeter (model HB, Brookfield Engineering Laboratories, Stoughton, MA, USA) equipped with a cone-plate spindle (CP52). The rheograms were recorded at different shear rates increasing from 0 to 25 s^−1^ for the upward curves, and then decreasing from 25 to 0 s^−1^ for the downward curves. All the rheograms were recorded within a total time of 240 s, in a thermostated cell at 25 °C. Data were collected and processed using the Rheocalc 32 software (Brookfield Eng., Middleborough, MA, USA). Rheological behavior was studied for each formulation and a viscosity value (in Pa·s) was recorded at a fixed rotational speed of 10 rpm for comparison.

Extensibility is defined as the area occupied by a given amount of sample to be subjected to a standard pressure between two glass plates. Extensibility tests were performed following the methods described by Campaña-Seoane [34]. Briefly, an extensometer consisting of two glass slides of 5 cm × 5 cm was used. One gram of the sample was deposited on the very center of the base plate and the second plate was disposed onto the sample. After a 3 min equilibrium time, the area of the formulation spread between the two plates was measured. The samples were compressed to uniform thickness by placing different weights (50, 100, 200, and 250 g) for 30 s. After each time, the area of the sample was determined. All experiments were conducted in triplicate and at room temperature.

Penetrometry experiments were carried out to determine the consistency of the formulations, using an Analis penetrometer equipped with a penetrating cone (Analis Instruments, Namir, Belgium), following the method described by European Pharmacopeia [35].

Microbiological quality was also determined for all the formulations. For the total bacteria recount, 0.5 g of each formulation was dissolved in 4.5 mL of peptone water. Serial dilutions were performed and seeded in Agar medium after incubation for two days at 37 °C. For the recount of coliform enterobacteria, 0.5 g of the formulations was dissolved in 4.5 mL of peptone water. After serial dilutions, the samples were seeded in Violet Red Bile Glucose (VRBG) medium after incubation for 24 h at 37 °C.

### 3.6. In Vitro Release Profile of Semi-Solid Formulations

A previously validated [36] PermeGear ILC-07 automated system (PermeGear, Riesgelsville, PA, USA) was used to perform the *in vitro* release studies. Briefly, the equipment incorporated seven in-line flow-through diffusion cells, made of Kel F, in which the donor and receptor chambers and the diffusion membrane placed over a support with a hole of 1 cm in diameter (having a diffusional area of 0.785 cm^2^) were clamped by threaded rods with adjustable locking nuts. Cellulose acetate dialysis membrane (average cutoff 12.000 Da) was selected as the diffusion membrane throughout all the experiments. All cells were placed in a cell warmer connected with a Haake-DC10 circulating bath (Gebruder Haake, Karlsruhe, Germany) to permit the system to be brought up to 37 °C. The receptor fluid consisting of phosphate buffered saline solutions (PBS pH 7.4 Sigma-Aldrich, Madrid, Spain) was pumped at a flow rate of 2 mL/h and collected in the receptor tubes in an Isco R^©^ Retriever IV fraction collector (Isco, Lincoln, NE, USA).

The evolution of the concentration of olive leaf extracts into the receiver chamber (Crec) of a flow-through diffusion cell *versus* time (*t*) was determined using the following equation:

Vrec × dCrec/dt = J × A − Frec × Crec,

where, Vrec is the volume of the receiver chamber, J the flux of drug through the membrane, A the diffusion area, and Frec the flow rate of receptor fluid. In this way, the term dCrec/dt could be easily estimated from the concentration *versus* time raw data.

The amounts of antioxidant components released from the different formulations containing olive leaf extracts were determined spectrophotometrically by the Folin–Ciocalteu method. All data were expressed as mean ± confidence interval. A *p*-value < 0.05 was considered to be statistically significant using the *t*-test between the two means for the unpaired data. Data analyses were conducted with SPSS software, version 14.0 (SPSS Science, Chicago, IL, USA).

## 4. Conclusions

Chitosan microspheres are adequate vehicles for the encapsulation of natural antioxidants from olive leaf extract. The methodology of the incorporation of the microspheres comprises its addition to the oil phase, guaranteeing a good dispersion. According to the *in vitro* release studies, chitosan microspheres loaded with olive leaf extract showed a progressive release. Likewise, it has been found that the incorporation of these microspheres in cosmetic moisturizers was effective because an adequate release of the active antioxidant ingredients from the microspheres was obtained.

The antioxidant effect was maintained in the long term in the developed formulations. The physico-chemical characteristics of the formulations were not negatively affected by the incorporation of the microspheres into the formulation. The antioxidant capacity of olive leaf extract was maintained in the long term, thus opening promising new perspectives for the application of chitosan microcapsules loaded with olive leaf extract in the cosmetic industry.

It was demonstrated that this microencapsulated system containing olive leaf extract and chitosan in semi-solid formulation is valid for applications in dermatology and cosmetics. We therefore propose a new system based on chitosan and olive leaf extracts with potential applications in the cosmetic dermatology and cosmetic industries.

## References

[1] Sailaja A.K., Awareshwar P.R. (2010). Chitosan nanoparticles as a drug delivery system. Res. J. Pharm. Biol. Chem. Sci..

[2] Gupta K.C., Jabrail F.H. (2006). Glutaraldehyde and glyoxal cross-linked chitosan microspheres for controlled delivery of centchroman. Carbohydr. Res..

[3] Caiqin Q., Huirong L., Qi X., Yi L., Juncheng Z., Yumin D. (2006). Water-solubility of chitosan and its antimicrobial activity. Carbohydr. Polym..

[4] He W., Guo X., Zhang M. (2008). Transdermal permeation enhancement of *N*-trimethyl chitosan for testosterone. Int. J. Pharm..

[5] Kosaraju S.L., D’ath L., Lawrence A. (2006). Preparation and characterization of chitosan microspheres for antioxidant delivery. Carbohydr. Polym..

[6] Belscak-Cvitanovic A., Stojanovic R., Manojlovic V., Komes D., Cindric I.J., Nedovic V., Bugarski B. (2011). Encapsulation of polyphenolic antioxidants from medicinal plant extracts in alginate-chitosan system enhanced with ascorbic acid by electrostatic extrusion. Food Res. Int..

[7] Harris R., Lecumberri E., Mateos-Aparicio I., Mengíbar M., Heras A. (2011). Chitosan nanoparticles and microspheres for encapsulation of natural antioxidants extracted from Ilex paraguariensis. Carbohydr. Polym..

[8] Chimi H., Cillard J., Cillard P., Rahmani M. (1991). Peroxyl and hydroxyl radical scavening activity of some natural phenolic antioxidants. J. Am. Oil Chem. Soc..

[9] Glampedaki P., Deuth V. (2006). Stability studies of cosmetic emulsions prepare from natural products such as wine, grape seed oil and mastic resin. J. Cosmet. Sci..

[10] Badiu D., Luque R., Rajendram R., Victor R. (2010). Effect of Olive Oil on the Skin. Olives and Olive Oil in Health and Disease Prevention.

[11] Desai P., Patlolla R.R., Singh M. (2010). Interaction of nanoparticles and cell-penetrating peptides with skin for transdermal drug delivery. Mol. Membr. Biol..

[12] Aranaz I., Mengibar M., Harris R., Miralles B., Acosta N., Calderon L., Sanchez A., Heras A. (2014). Role of Physicochemical Properties of Chitin and Chitosan on their Functionality. Curr. Chem. Biol..

[13] Aranaz I., Mengíbar M., Heras A. (2009). Functional Characterization of Chitin and Chitosan. Curr. Chem. Biol..

[14] Montreau F.R. (1972). On the analysis of total phenolic compounds in wines by the Folin-Ciocalteu method. Connaiss. Vigne Vin.

[15] Sifaoui I. (2014). Activity of olive leaf extracts against the promastigote stage of *Leishmania* species and their correlation with the antioxidant activity. Exp. Parasitol..

[16] Stulzer H.K., Tagliari M.P., Parize A.L., Silva M.A.S., Laranjeira M.C.M. (2009). Evaluation of cross-linked chitosan microparticles containing acyclovir obtained by spray-drying. Mater. Sci. Eng..

[17] Fang Z., Bhandair B. (2010). Encapsulation of polyphenols—A Review. Trends Food Sci. Technol..

[18] Argüelles-Monal W., Peniche-Covas C. (1988). Study of the Interpolyelectrolyte Reaction between Chitosan and Carboxymethyl Cellulose. Die Makromol. Chem. Rapid Commun..

[19] Bocourt M., Argüelles W., Cauich J.V., Bada N., Peniche C. (2011). Interpenetrated chitosan-poly (acrylic acid-co acrylamide) hydrogels. Synthesis, characterization and sustained protein release studies. Mater. Sci. Appl..

[20] Osman Z., Arof A.K. (2003). FTIR studies of chitosan acetate based polymer electrolytes. Electrochim. Acta.

[21] Bunjes H., Unruh T. (2007). Characterization of lipid nanoparticles by differential scanning calorimetry, X-ray and neutron scattering. Adv. Drug Deliv. Rev..

[22] Ioelovich M. (2014). Crystallinity and Hydrophility of Chitin and Chitosan. J. Chem..

[23] Hidalgo C., Fernández M., Nieto O.M., Paneque A.A., Fernández G., Lópiz J.C.L. (2009). Estudio de quitosanos cubanos derivados de la quitina de la langosta. Rev. Iberoam. Polím..

[24] Yenilmez E., Başaran E., Yazan Y. (2011). Release characteristics of vitamin E incorporated chitosan microspheres and *in vitro*-*in vivo* evaluation for topical application. Carbohydr. Polym..

[25] Bernkop-Schnürch A. (2005). Mucoadhesive polymers: Strategies, achievements and future challenges. Adv. Drug Deliv. Rev..

[26] Rosen R.M. (2005). Delivery System Handbook for Personal Care and Cosmetic Products: Technology, Applications and Formulations.

[27] Popa M., Aelenei N., Popa V.I., Andrei D. (2000). Study of the interactions between polyphenolic compounds and chitosan. React. Funct. Polym..

[28] Ramírez-Moreno E., Córdoba-Díaz M., de Cortes Sánchez-Mata M., Marqués C., Isabel Goñi I. (2015). The addition of cladodes (*Opuntia ficus indica* L. Miller) to instant maize flour improves physicochemical and nutritional properties of maize tortillas. LWT—Food Sci. Technol..

[29] Prudencio I.D., Prudencio E.S., Gauche C., Barreto P., Bordignon-Luiz M.T. (2008). Flow properties of petit Suisse cheeses use of cheese whey as a partial milk substitute. Ital. J. Food Sci..

[30] Di Mambro V.M. (2005). Assays of physical stability and antioxidant activity of a topical formulation added with different plant extracts. J. Pharm. Biomed..

[31] Benzie I.F.F., Strain J.J., Packer L. (1999). Ferric Reducing/Antioxidant Power Assay: Direct Measure of Total Antioxidant Activity of Biological Fluids and Modified Version for Simultaneous Measurement of Total Antioxidant Power and Ascorbic Acid Concentration. Methods in Enzymology.

[32] Pulido R., Bravo L., Saura-Calixto F. (2000). Antioxidant activity of dietary polyphenols as determined by a modified ferric reducing/antioxidant power assay. J. Agric. Food Chem..

[33] Harris R., Paños I., Acosta N., Heras A. (2008). Preparation and characterization of chitosan microspheres for controlled release of tramadol. J. Control. Release.

[34] Campaña-Seoane P.A. (2014). Bioadhesive emulsions for control release of progesterone resistant to vaginal fluids clearance. Int. J. Pharm..

[35] (2008). Measurement of Consistency by Penetrometry, 267. European Pharmacopoeia 7.0.

[36] Córdoba-Díaz M., Nova M., Elorza B., Córdoba-Díaz D., Chantres J.R., Córdoba-Borrego M. (2000). Validation protocol of an automated in-line flow-through diffusion equipment for *in vitro* permeation studies. J. Control. Release.

